# Structural determination of the HIV-1 Variable Region 3 epitope of antibody 19b

**DOI:** 10.1128/jvi.02139-25

**Published:** 2026-07-22

**Authors:** Susan K. Fetics, Ariha Mehta, Nathan I. Nicely, Chun-Hsing (Josh) Chen, Jared Lindenberger, Priyamvada Acharya

**Affiliations:** 1 Duke Human Vaccine Institute, Duke University 188743, Durham, North Carolina, USA; 2 University of North Carolina at Chapel Hill 2331, Chapel Hill, North Carolina, USA; 3 Department of Surgery, Duke University 160976, Durham, North Carolina, USA; 4 Department of Biochemistry, Duke University 219047, Durham, North Carolina, USA; University Hospital Tübingen, Tübingen, Germany

**Keywords:** crystal structure, non-neutralizing, monoclonal antibody 19b, cradle binding mode, V3 epitope, HIV-1 envelope

## Abstract

The HIV-1 Envelope (Env) in its pre-receptor “closed” conformation is targeted by broadly neutralizing antibodies (bnAbs), while its receptor-bound “open” conformation exposes immunodominant epitopes targeted by non-neutralizing antibodies. A human immunoglobulin G (IgG) monoclonal antibody (mAb), 19b, binds an Env third variable (V3) loop epitope that is only exposed in the open Env conformation. Despite widespread use of 19b to detect the open Env conformation in immunoassays, its epitope has not yet been structurally defined. Here, we determine crystal structures of ligand-free and V3 peptide-bound 19b Fab to visualize details of this interaction. 19b utilizes both its heavy and light chains to interact with the V3 loop. The 5-residue heavy-chain complementarity-determining region (CDR H3) forms a hydrophobic binding pocket to bind V3 residues. 19b adopts a cradle-binding mode, with its CDRH1, CDRL2, and CDRL3 mediating interactions with the V3 regions flanking the conserved GPGR/Q motif, while making only limited contacts with the GPGR arch region. Our high-resolution structures elucidate the epitope, binding mode, and the structural basis for the broad reactivity of 19b, thereby filling a gap in our knowledge of a widely used reagent in immunoassays.

**IMPORTANCE:**

Here, we determine high-resolution crystal structures of 19b, an antibody that binds the receptor-bound “open” conformation of the HIV-1 envelope (Env) protein at an epitope located within its third variable (V3) loop. Despite widespread use of 19b to detect the open Env conformation in immunoassays, its epitope has not yet been structurally defined. Our high-resolution structures, by elucidating the epitope, binding mode and the structural basis for the broad reactivity of 19b, fill a gap in our knowledge of a reagent that is widely used in immunoassays.

## INTRODUCTION

Human immunodeficiency virus type 1 (HIV-1) expresses a single viral protein, called envelope (Env), on its cell surface. As a result, Env is the sole target for HIV-1 neutralizing antibodies (1). The Env glycoprotein has five variable loop regions (V1–V5), of which the V3 loop harbors conserved binding motifs at its base and a GPGR/Q motif at its apex that interacts with the coreceptor, CCR5 or CXCR4 (Fig. S1). The sequence of V3 predicts co-receptor specificity, while its conformation determines co-receptor binding and virus infection (2, 3).

**Fig 1:**
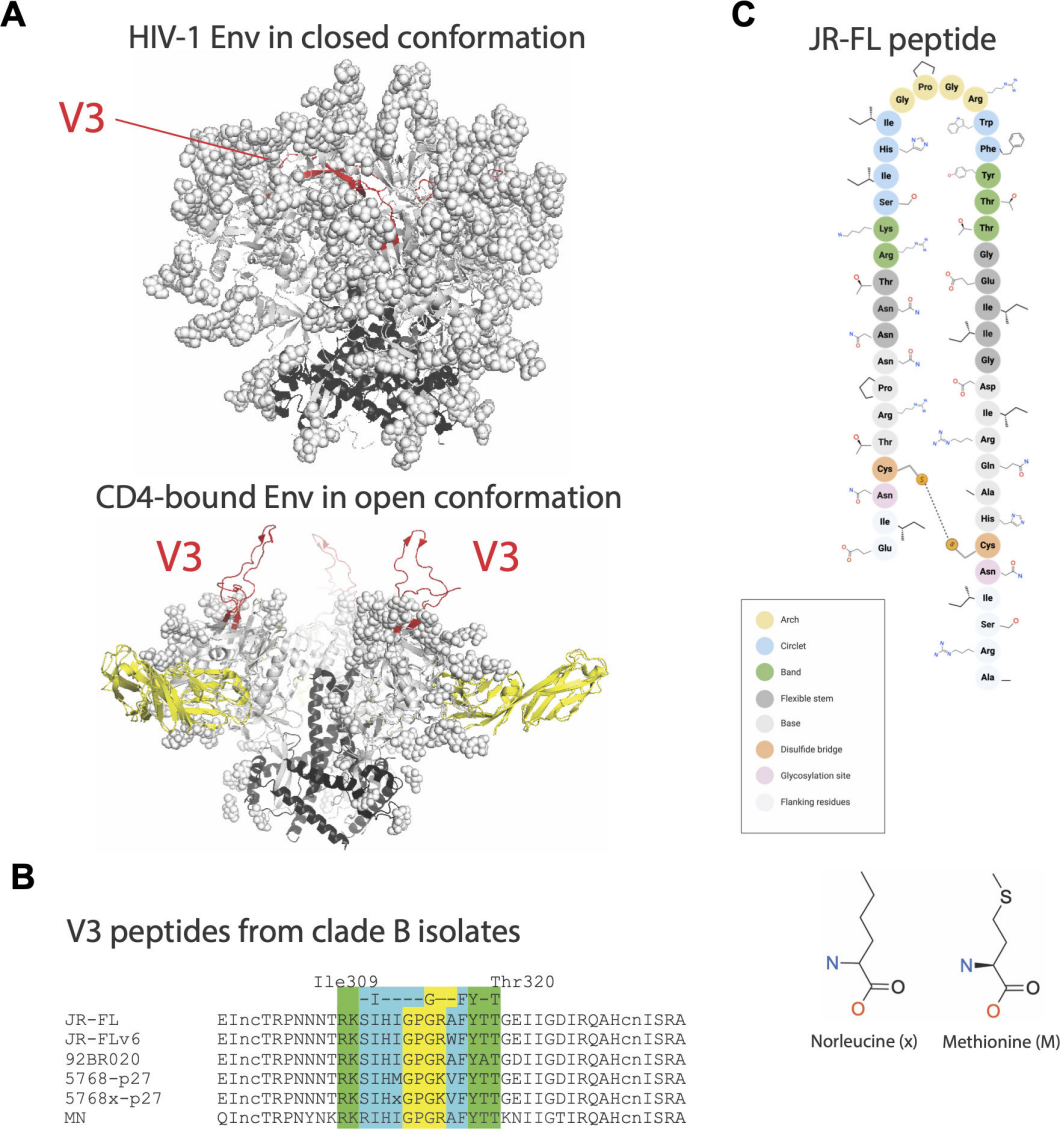
Crystal structure of 19b Fab in the presence of peptide V3 JR-FL. (**A**) HIV-1 Env shown in cartoon representation in the closed (top) and CD4-bound open (bottom) conformations, with gp41 colored black, gp120 gray, and the V3 peptide colored red. Glycans are shown as spheres. (**B**) V3 peptide sequences used in this study and of isolate MN. (**C**) (Top) A schematic diagram of the JR-FL V3 peptide showing the side chains for each residue. (Bottom) Chemical structures of norleucine and methionine.

In the “closed” Env conformation prior to receptor binding, the V3, shielded by its interactions with the V1V2 regions, is inaccessible for co-receptor binding. After Env engages the CD4 receptor, conformational changes occur in Env that are collectively termed Env “opening.” In the open Env conformation, the V3 region extends from the Env core, no longer interacting with V1V2, with the extended V3 available for coreceptor binding. Antibodies that target the V3 crown in the open Env conformation show strain-specific neutralization and are present in most persons infected with HIV-1 (Fig. 1A and S1 ) (3–13).

The V3 region is ~50 Å in length and consists of a conserved base (residues 296–300 & 326–331), a flexible stem (residues 301–305 & 321–325) and a β-hairpin loop crown (residues 306–320, Fig. 1B). A disulfide bond between residues C296 and C331 at the base stabilizes the V3 region (2, 13, 14) (Fig. 1C). The crown can be further broken down into three regions: the band (306–308 & 318–320), the circlet (309–311 & 316–317), and the arch (GPGR/Q at 312–315), with the band and circlet regions flanking the GPGR/Q arch (11).

Human IgG monoclonal antibody N701.9b, commonly known as 19b, was isolated from an asymptomatic person with HIV-1 (15, 16). Alanine scanning of V3 residues 308 to 318 showed that Env binding to 19b occurs at residues 313–318 within the crown (15). Further amino acid substitution analysis revealed a V3 motif that directly affects Env interaction with 19b: -I----G--FY-T, where a dash denotes any residue and a letter denotes the residue at which contact must occur. These residues span the V3 β-hairpin turn, occupying the arch, circlet, and band regions. Despite the diversity of V3 residues in HIV-1 strains, 19b binding is observed for HIV-1 subtypes A, B, C, E, and F, illustrating inter-clade cross-reactivity (16, 17). As a poorly neutralizing antibody, 19b only neutralizes Tier 1 isolates (18). Early studies indicated that the Env glycan shield can prevent accessibility of the 19b epitope (19).

Here, to define the 19b epitope at the atomic level, we determined crystal structures of the 19b antigen-binding fragment (Fab) in complex with aglycone V3 peptides from HIV-1 clade B isolates JR-FL, 92BR020, and 5768-p27. Previous studies showed that 19b neutralized the 5768-p27 and 92BR020 isolates with IC_50_ of 3.2 µg/mL and 21.5 µg/mL, respectively, while no neutralization of JR-FL was observed at the concentrations tested (IC_50_ > 50 µg/mL) (20). We also determined the structure of the ligand-free 19b Fab. 19b engages the V3 in the cradle binding mode (abbreviated as V3C), making contacts with hydrophobic core residues in the circlet and band regions, with minimal contacts with the GPGR/Q arch. The cradle binding mode adopted by 19b is distinguished from the ladle binding mode (V3L), in which interactions occur with the GPGR arch. The 5-residue complementarity-determining region (CDR) 3 of the 19b heavy chain (CDR H3) creates a pocket for V3 binding. The dominant interactions are established between the band and circlet regions of V3 and the CDR 2 and 3 of the light chain (L2 and L3) and the CDR H1.

## RESULTS

### Crystallization of 19b Fab with HIV-1 Env V3 peptides

For co-crystallizing with 19b Fab, we synthesized V3 peptides from clade B HIV-1 isolates JR-FL, 92BR020, and 5768-p27 (Fig. 1B). A modified version of the JR-FL V3 peptide, called JR-FL.v6 V3 peptide here, was used (21). In addition to the native 5768-p27 V3 peptide, a second peptide, 5768x-p27, was synthesized in which Met311 was substituted with norleucine to prevent unwanted oxidation (Fig. 1C). The synthetic peptides spanned 43 residues (295–337), including the V3 base, crown, and stem regions, with a disulfide bond between Cys297 and Cys332.

**Fig 2:**
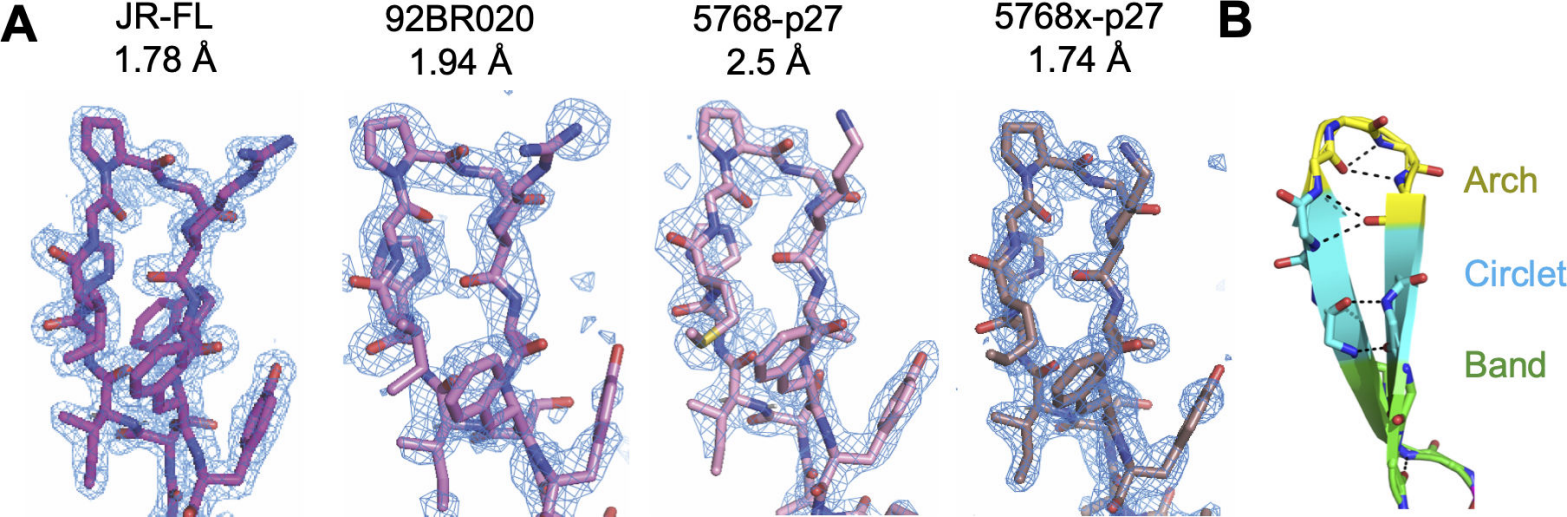
Crystal structure of diverse clade B V3 peptides bound to antibody 19b. (**A**) Difference density (Fo-Fc map) represented as a blue mesh, fitted with coordinates in stick representation of the V3 peptides from JR-FL (magenta), 92BR020 (violet) 5768-p27 (pink), and 5768x-p27 (dark violet). Fo-Fc map is contoured at 3σ. (**B**) The 19b-bound JR-FL peptide main chain is shown in cartoon representation and colored by the arch (yellow), circlet (cyan), and band (green) regions. Residue main chains are shown as sticks and colored by atom type (nitrogen—blue, oxygen—red). Dashed lines indicate hydrogen bonds.

19b Fab was crystallized, ligand-free, and bound to four aglycone V3 peptides. Each crystal data set contains one molecule in the asymmetric unit with the space group P2_1_2_1_2_1_ and similar unit cell parameters (Tables S1 to S4; Fig. 2). For this study, residue numbering for 19b follows the Kabat and Wu convention, and for the V3 peptides follows the HXB2 number scheme (22). In the 19b-bound V3 peptide structures, electron density was present for the central 15–19 residues of V3 flanking the GPGR/K arch, where the peptide model was built (Fig. 2A). All the bound V3 peptides showed similar β-hairpin loop conformation, exhibiting a type-II β turn at the GPGR/K arch residues 312–315, stabilized by hydrogen bonds mediated by backbone residues (Fig. 2B). Superposition of the V3 peptides from the different structures yielded Cα RMSD values of ~0.2 Å (Table S3).

### Details of V3 peptide interactions with 19b

**Fig 3:**
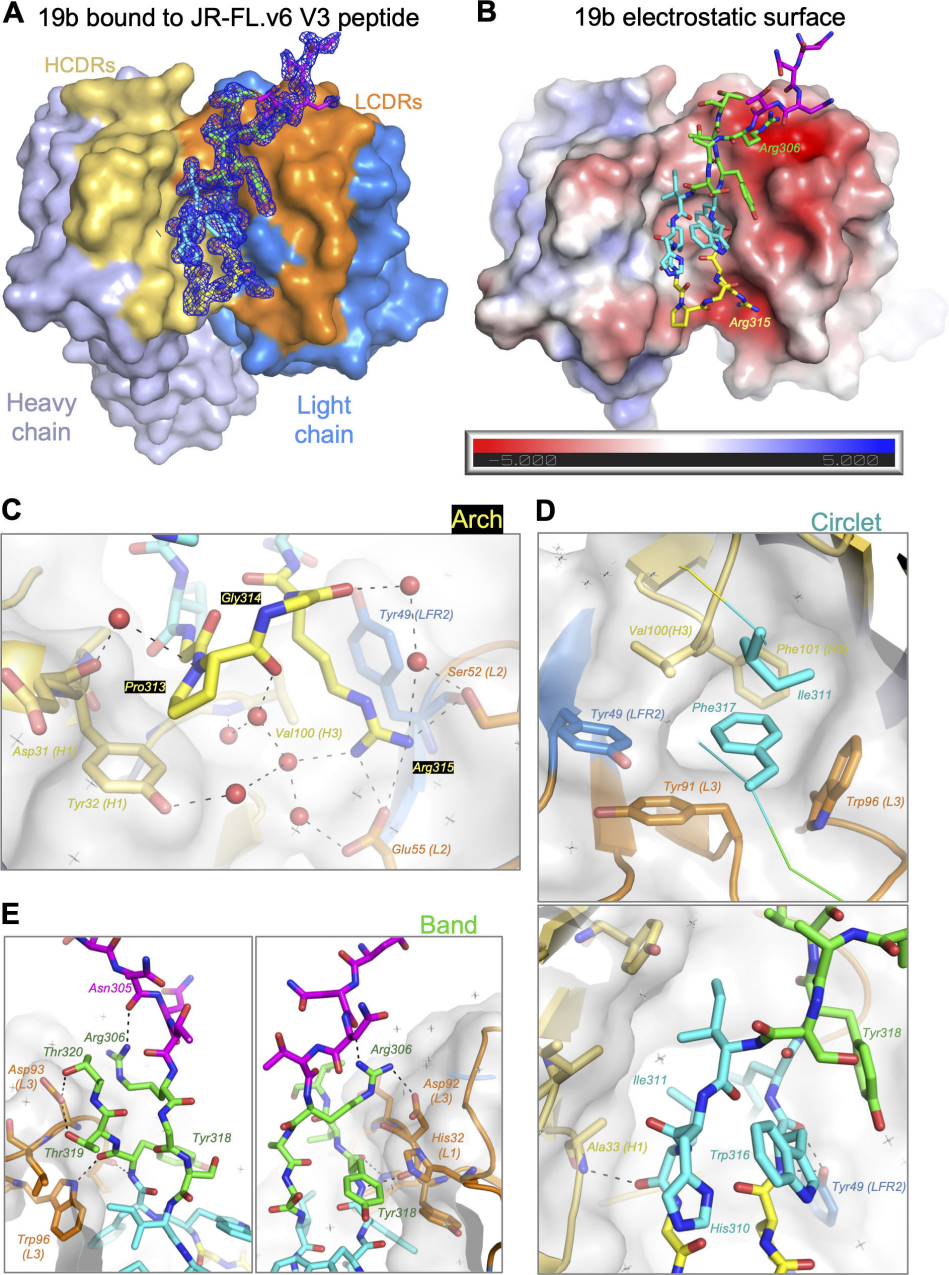
Crystal structure of 19b Fab in complex with the JR-FL.v6 V3 peptide. (**A**) 19b Fab, shown in surface representation, bound to the JR-FL V3 peptide, shown in stick representation. 19b Fab heavy chain is colored light blue, and light chain is marine. The CDR loops are colored: L1/L2/(L3 (orange), H1/H2/H3 (yellow-orange). The cradle motif of epitope interaction for V3 and 19b Fab is observed. Electron density for the V3 peptide of JR-FL is shown as a blue mesh, with the 2Fo-Fc map contoured at 1σ. JR-FL V3 is shown in stick representation with residues colored as follows: band (green), circlet (cyan), arch (yellow). Residues outside these three regions in the V3 loop are colored magenta. (**B**) Electrostatic potential surface of 19b Fab with bound V3 peptide shown as sticks and colored similarly as in panel A. (**C through E**) Details of interactions between 19b Fab with V3 residues in the (**C**) Arch, (**D**) Circlet, and (**E**) Band regions. Two ~180° rotated views are shown to illustrate the interactions of the Band region with 19b. 19b is shown in cartoon representation with V3-interacting residues shown as sticks. An overlaid, light-gray, transparent surface is also shown for 19b. Dashed lines indicate hydrogen bonds. Key water molecules are shown as red spheres. Other waters are visible as light gray “+” signs.

To visualize the interactions between 19b and the HIV-1 Env V3 region, we first studied JR-FL.v6 V3 peptide-bound 19b Fab complex (Fig. 3; Fig. S2 and Table S4). Both heavy and light chains of 19b interacted with V3, with the 19b CDRs contacting the V3 crown at the interface between the antibody heavy and light chains (Fig. 3A and B; Table S4). The 19b light chain contributed a more electronegative surface compared to the heavy chain (Fig. 3B). Electrostatic complementarity was observed in the interaction, with the more electropositive surface of the V3 peptide interacting with the electronegative region of the 19b paratope (Fig. 3B). The V3 crown interacted with four of six 19b CDRs: L2, L3, H1, and H3 (Fig. 3C through E). All three regions of the V3 crown—the arch, circlet, and band—contacted 19b (Fig. 3C through E). Characteristic of the cradle-binding motif, the GPG turn of the GPGR arch motif was solvent-exposed (Fig. S2). While this region did not make direct contacts with 19b, hydrogen bonds mediated by a solvent network were observed with heavy chain residues Asp31 (H1), Tyr32 (H1), and Val100 (H3) and light chain residues Ser52 and Glu55 (L2) (Fig. 3C). A salt bridge was observed between the guanidine side chain of Arg315 in the GPGR motif and the 19b residue Glu55 (L2). The 19b CDR L2 residue Glu55 contributes to the electronegative character of the portion of the paratope coming from the 19b light chain (Fig. 3B). The side chain of Arg315 was further stabilized by stacking against the side chain of Tyr49 (L2) (Fig. 3C).

A key interaction was mediated by the V3 circlet residue Phe317 that inserts its aromatic side chain into a pocket lined by hydrophobic and aromatic residues—Tyr91 (L3), Trp6 (L3), Tyr49 (LFR2), Val 100 (H3), Phe101 (H3), and V3 residue Ile312 (Fig. 3D). The central role of residue Phe317 in V3 binding to 19b was recognized previously (20). Other circlet interactions include hydrogen bonds mediated by the main chain of residues 311 and 316. The main chain carbonyl of Ile311 engages in a hydrogen bond with the main chain nitrogen of Ala33 (H1). The 19b light chain residue Tyr49 (LFR2) utilizes its side chain hydroxyl to engage in hydrogen bonds with the main chain of residue 316. Residue 316 in the JRFL.v6 V3 peptide is the site of the Ala to Trp substitution that was incorporated to stabilize the pre-fusion, closed Env conformation (21). The lack of interaction of the Trp316 side chain with the antibody explains why binding of the V3 peptide to 19b is compatible with the substitution of Ala316 in JR-FL Env with the bulkier Trp. In our structure, the Trp side chain stacks against the side chain of the V3 residue His312.

The band segment of the V3 crown uses both main chain and side chain contacts to interact with 19b (Fig. 3E). The main chain carbonyl of V3 residue Tyr318 hydrogen bonds with the 19b residue Trp96 (L3), while the Asp93 (L3) side chain engages in hydrogen bonds with the side chains of the V3 residues Thr319 and Thr320. An intra-V3 hydrogen bond was observed between the side chain of residue Arg306 and the main chain carbonyl oxygen of Asn305. This interaction brings the positively charged side chain of Arg306 into proximity with the electronegative surface contributed by the light chain of 19b and facilitates a salt-bridge interaction with Asp92 (L3) (Fig. 3B and E).

In summary, the crystal structure of 19b Fab bound to the JRFL.v6 V3 peptide provides atomic-level visualization of the V3-antibody interactions and provides a structural basis for the previously observed roles of critical residues involved in the interaction.

### Recognition of diverse V3 peptides by 19b

**Fig 4:**
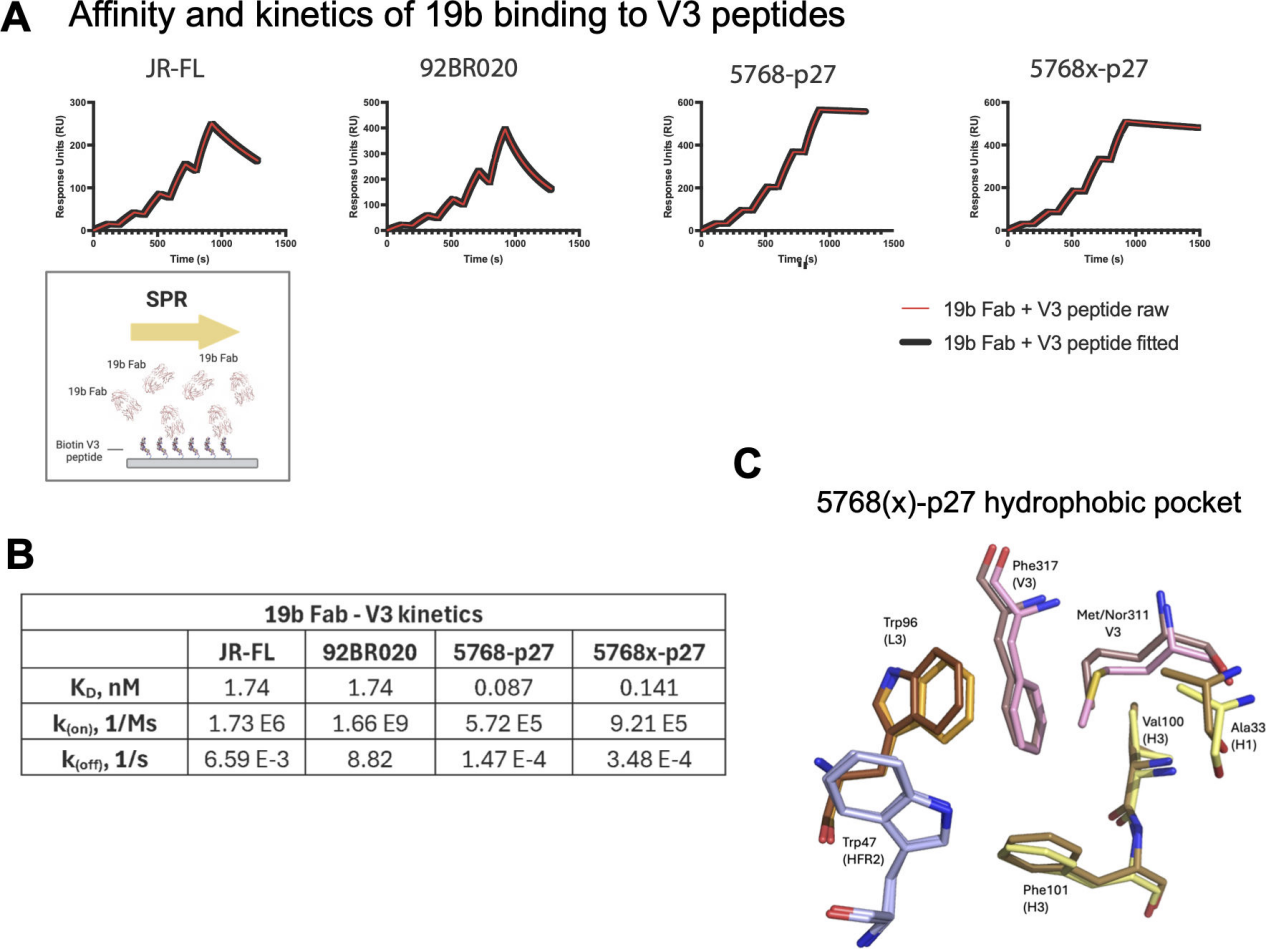
19b binding to diverse V3 peptides. (**A**) SPR sensorgrams are shown for 19b Fab binding to V3 peptides (left to right), JR-FL, 92BR020, 5768-p27, and the 5768x-p27 V3 peptide, in which the methionine at position 311 was replaced with a norleucine in the 5768-p27 peptide. The reference-subtracted sensorgrams are shown as red lines in the graphs, with the black lines indicating the fit of the data to a 1:1 Langmuir binding model. The inset is a schematic of the experimental design showing the V3 peptide captured onto a streptavidin surface via its biotin tag, with the 19b Fab flowing over the surface to measure binding. (**B**) Table listing the affinity and kinetics of binding of each V3 peptide to the 19b Fab. (**C**) The hydrophobic pocket into which V3 Phe 317 is buried in its 19b-bound state is shown for the 19b complexes with the 5768-p27 and 5768x-p27 V3 structures. For the 5768-p27 structure, V3 is colored pink, LCDR3 bright orange, HCDR1 and HCDR3 pale yellow, and for the 5768x-p27 structure, V3 is colored dark violet, LCDR3 brown, HCDR1 and HCDR3 sand.

To obtain quantitative measures of the affinity and kinetics of the interaction between 19b and the V3 peptides, we performed surface plasmon resonance (SPR) binding assays (Fig. 4A and B). The 19b Fab bound the JR-FL.v6 and 92BR020 V3 peptides with a similar affinity of 1.74 nM (Fig. 4B). The V3 sequences of these isolates differ by two residues at positions 316 and 319 (Fig. 1B). The 5767-p27 V3 peptides showed ~20 fold tighter binding (*K*_D_ = 0.087 nM), with a substantially slower off-rate compared to the JR-FL.v6 and 92BR020 V3 peptides. Therefore, despite our crystal structures showing remarkable similarity in the 19b-bound conformations of V3 peptides from different HIV-1 isolates (Fig. 2A), we observed considerable variation in the affinity and kinetics of binding of the V3 peptides to 19b. The differences in affinity observed between the 92BR020 and the 5767-p27 V3 peptides were consistent with the differences in neutralization of the corresponding pseudoviruses by 19b (20). On the other hand, JR-FL was not neutralized by 19b even though its V3 peptide bound with similar affinity as the 92BR020 V3 peptide, consistent with the JR-FL isolate predominantly being in the closed conformation with the V3 crown epitope occluded and inaccessible to 19b binding.

In the 5768(x)-p27 V3 peptide-bound 19b Fab structures, the methionine (in 5768-p27) and the non-standard norleucine (in 5768x-p27) replace Ile at position 311 to participate in forming the hydrophobic pocket that buries the V3 Phe317 residue (Fig. 4C). The preference for a Met at V3 position 311 for its interaction with 19b was discussed previously (20). Indeed, this is reflected in the ~10-fold higher affinity of the 19 b Fab for the 5768-p27 V3 peptides compared to the JR-FL and 92BR020 V3 peptides. The V3 of 5768(x)-p27 harbors a lysine at position 315 within the arch in lieu of arginine in JR-FL and 92BR020, thereby weakening the interaction observed between V3 position 315 and Glu55 (L2) of 19b as seen in JR-FL and 92BR020 (Fig. 3C), confirming the non-essential nature of this arch interaction and further supporting the circlet/band “cradle” binding motif between 19b Fab and Env V3 peptide (Fig. S1).

### Crystal structure of ligand-free 19b Fab

**Fig 5:**
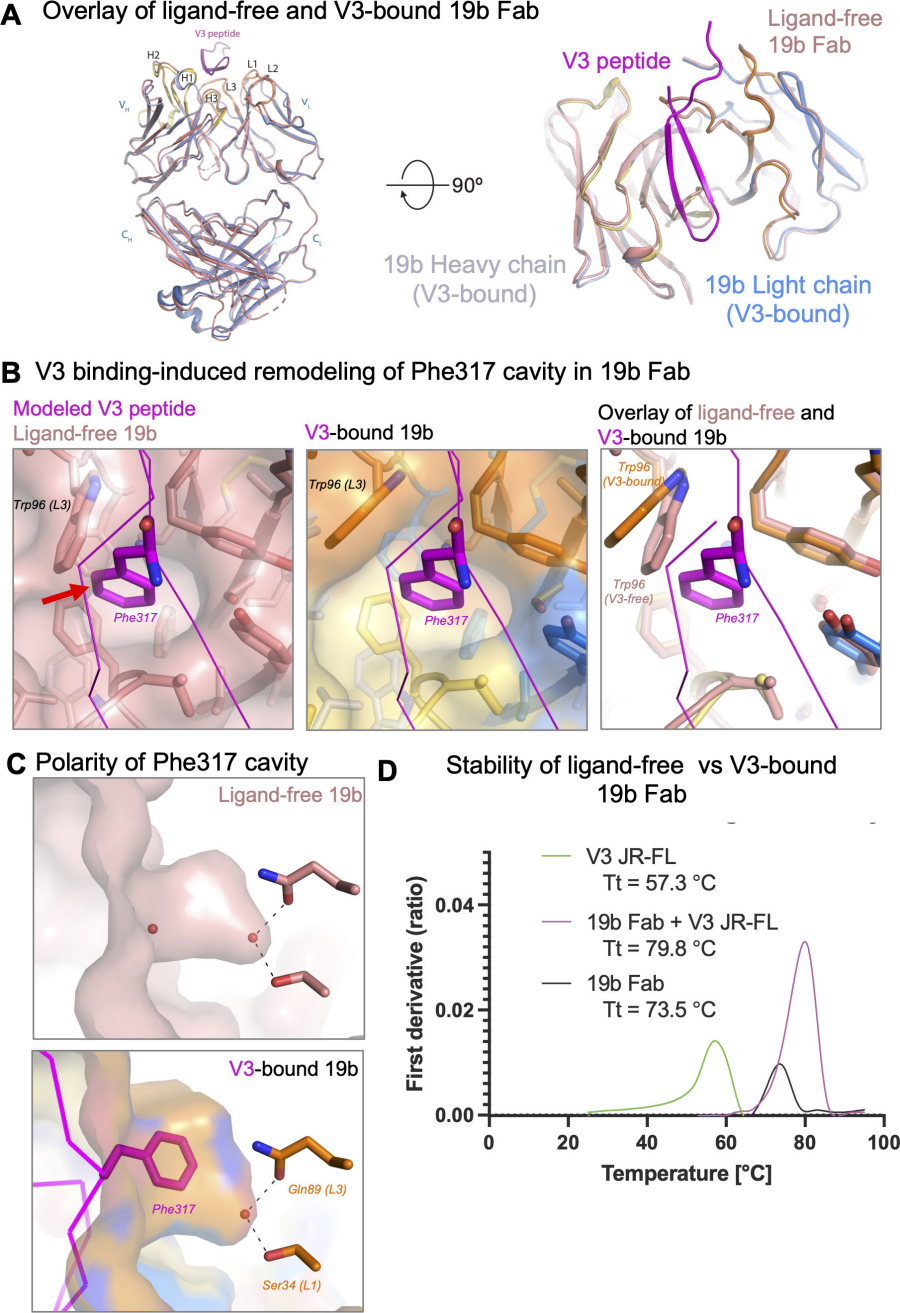
Crystal structure of ligand-free 19b Fab. (**A**) Crystal structure of the ligand-free 19b Fab (colored salmon) overlaid on the structure of the JR-FL.v6 V3 peptide (colored magenta)-bound 19b Fab (heavy chain colored light blue, light chain marine, HCDRs colored sand and LCDRs colored orange). Two 90° rotated views are shown. (**B**) Comparison of the cavity into which the V3 residue Phe317 side chain buries. Left. Ligand-free 19b structure shown as a transparent salmon surface and in cartoon representation with side chains shown as sticks. Shown in magenta ribbon representation is the V3 peptide with the Phe317 residue shown in stick representation. The V3 peptide is modeled onto the ligand-free structure based on the overlay shown in panel A. The red arrow indicates a clash between the inserted Phe317 phenyl group and the cavity wall lined by the Trp96 (L3) side chain. Middle. JR-FL.v6 V3 peptide-bound 19b, with the antibody structure shown as a transparent surface, and in cartoon representation with side chains shown as sticks. Right. Overlay of ligand-free and V3-bound structures zoomed into the Phe317 binding region. A rotameric shift in the Trp96 (L3) side chain enlarges the cavity, allowing the phenyl group of the V3 residue Phe317 to insert. (**C**) Side views of the Phe317 insertion cavity. Top. Ligand-free 19b. Bottom. V3-bound 19b. Water molecules in the cavity are shown as red spheres. Dashed lines indicate hydrogen bonds. (**D**) Differential Scanning Fluorimetry of ligand-free 19b Fab (black), V3-bound 19b Fab (magenta), and the V3 peptide (green).

To study V3 peptide binding-induced changes in 19b, we solved the crystal structure of V3-free 19b and compared it to the structure of 19b bound to the JR-FL V3 peptide (Fig. 5). We observed close agreement between the two structures with Cα RMSD value of 0.807 Å (Fig. 5A). The six CDR main chains adopt the same conformation in the ligand-free 19b when compared to 19b in complex with V3 JR-FL. The Cα RMSD values for all six CDRs between the ligand-free 19b Fab and 19b Fab co-crystallized with V3 JR-FL structures are below 0.3 Å (Table S3). Ligand-free 19b CDRs L2, L3, and H1 have Cα RMSD values of 0.143 Å, 0.117 Å, and 0.069 Å, respectively, relative to the JR-FL V3—19b structure (Table S3). Around the 19b L2 region, two water molecules were observed to form hydrogen bonds with the main chain carbonyls of residues Ser52 and Val51 (Fig. S3 ). Testing whether the protein structure prediction algorithm, AlphaFold (23), can recapitulate the V3 peptide-antibody binding interface observed in the crystal structures, notable divergence was observed in the main chain at CDR L2 where the Cα RMSD is 1.456 Å between the experimental and AlphaFold-modeled structures, impacting the observed interactions of the V3 peptide with the L2 region (Fig. S3). L2 Residue Ser52 in the AlphaFold model was not within hydrogen bonding distance of Arg315 from V3 JR-FL although the interaction between Glu55 (L2) and Arg315 (V3) was retained. These observations suggest a structural role for these waters in stabilizing the experimentally observed conformation of the L2 region.

Although the main chain configurations of the ligand-free 19b Fab and the V3-bound 19b Fab were similar, an important structural shift was observed in the “Phe317 cavity,” the binding pocket that is lined by 19b heavy and light chain residues, and into which the V3 residue Phe317 inserts (Fig. 5B). In the V3 peptide-bound structure, a rotameric shift in the light chain Trp96 (L3) side chain enlarges the cavity, allowing the phenyl side chain of the V3 Phe317 residue to insert. In the ligand-free 19b structure, we observed two water molecules in the Phe317 insertion cavity—one near the mouth of the cavity and another placed deeper into the cavity, making hydrogen bonds with light chain residues Ser34 (L1) and Gln89 (L3) (Fig. 5C). In the V3-peptide-bound structure, the water molecule near the mouth of the cavity was displaced by the binding ligand, but the second water molecule that was located deeper in the cavity was retained. Previous studies have shown that Phe317 can be substituted by Ile, Leu, Val, or Tyr and still retain 19b binding (20). It is possible that when substituted by Tyr, the Tyrosine side chain hydroxyl displaces the water molecule from the cavity and takes its place, forming hydrogen bond with Ser34 (L1) and Gln89 (L3). Our structures, thus, reveal a malleable cavity that can adjust its shape to accommodate the size, shape, and polarity of the incoming residue.

Using Differential Scanning Fluorimetry (DSF), a technique that monitors changes in intrinsic protein fluorescence upon application of a thermal ramp, we observed an increase in the stability of the 19b Fab when it was bound to the V3 peptide compared to the ligand-free 19b Fab (Fig. 5D). The ligand-free 19b Fab showed a transition temperature of 73.5°C, which increased by 6.3°C resulting in a transition temperature of 79.8°C when the 19b Fab was bound to the JR-FL.v6 V3 peptide.

Taken together, these results show how the HIV-1 Env V3 engages with the 19b antibody and stabilizes it, with minimal changes to the 19b main-chain residues and elucidate the mechanism by which a key binding cavity for the V3 residue 317 adapts to V3 diversity and accommodates different amino acids.

## DISCUSSION

The HIV-1 Env V3 region facilitates binding to the GPCR coreceptor, either CCR5 or CXCR4, which constitutes a key step in HIV-1 entry into host cells. Despite variability among HIV-1 isolates, key V3 loop motifs are conserved, enabling coreceptor binding. The V3 region is targeted by two categories of antibodies, distinguished by the V3 region they bind and the Env conformation they target. The first category includes broadly neutralizing antibodies that bind an epitope accessible on pre-receptor and closed Env and involves residues at the V3 base (24–28). The second category, to which 19b belongs, binds to the tip of V3 and its flanking regions, which are only accessible in the open Env conformation. These antibodies are non-neutralizing or show limited, strain-specific neutralization (4–17).

In the closed, neutralization-resistant conformation of HIV-1 Env, the 19b binding site is occluded and becomes available only upon CD4-induced Env opening (29) (Fig. S1). Once Env has bound CD4, however, the proximity to the host membrane would render antibody access sterically unfavorable. As a result, despite their strong binding, 19b-like antibodies are non-neutralizing or only neutralize isolates that readily sample the open conformation, even in the absence of receptor binding. 19b-like antibodies are found in most individuals with HIV-1. Due to the conformation-selective nature of their binding, these antibodies likely drive intra-host viral evolution toward more neutralization-resistant, closed conformations.

Stabilizing the closed Env conformation that only binds bnAbs and not non-neutralizing antibodies like 19b has been a longstanding goal of HIV-1 vaccine development, with several strategies developed to engineer a closed Env (30–32). 19b has been extensively used in immunoassays to detect open Env conformations (29, 31–34). While the general location of the 19b epitope was known, this study now provides high-resolution structures that confirm a cradle-binding mode for 19b (Fig. S2), thereby defining the 19b epitope at atomic resolution and elucidating the structural basis for its broad reactivity across diverse HIV-1 isolates. Combining the structural observations with the SPR assays revealed differences in the kinetics and affinity of diverse V3 peptide engagement with 19b, even though the final bound poses of the V3 peptides were almost identical, providing a glimpse into how 19b accommodates V3 sequence and conformational diversity.

Although targeting the closed Env conformation is a widely pursued goal in HIV-1 vaccine development, several key findings suggest that this paradigm may warrant reconsideration (5, 33, 35). The observation that 19b-like antibodies can neutralize some “difficult-to-neutralize” Tier 2 isolates indicates that these isolates sample open conformations in their native state (5). Indeed, it is now recognized that incompletely closed Env conformations in some viruses may be associated with resistance to bnAbs (33). Additionally, Fc-mediated effector functions have been demonstrated for 19b-like V3 antibodies (36). Thus, preventive and therapeutic approaches should consider the role of these antibodies.

Our studies provide the structural basis for previously published reports demonstrating the importance of specific V3 residues in 19b binding. A key role was assigned to the V3 residue 317 in previous studies (16). We found that the aromatic side chain of Phe317 was inserted into the mouth of a cavity lined by residues from the heavy and light chains (Fig. 5). This “Phe317” cavity is reminiscent of the “Phe43” cavity that engages residue Phe43 of CD4 at the junction of the inner and outer domains of the HIV-1 Env gp120 subunit (37). Like the gp120 “Phe43” cavity, the “Phe316” cavity of 19b is malleable and can accommodate other hydrophobic/aromatic side chains in place of a Phenylalanine. Both cavities also exhibit polarity through internal water molecules that can accommodate polar side-chain moieties within the cavities. Thus, alongside the gp120 “Phe43” cavity, the 19b “Phe317” cavity is another example of how a single phenylalanine residue can play a dominant role in ligand binding through insertion of its aromatic side chain into an interfacial cavity to glue together different domains or subunits of the protein that they are binding to.

Through combined insights from high-resolution experimental structural determination and protein structure prediction, we identified water molecules in the structure of the 19b Fab that could be critical for maintaining the conformation of the CDR L2 region and hence likely to be important for function. In the absence of these waters, AlphaFold failed to predict the structure of the CDR L2 region correctly (Fig. S3). Thus, our studies provide mechanistic insights into the role of structural waters and highlight a blind spot in modern protein prediction algorithms that don’t consider waters in their predictions. Close integration of these powerful protein structure prediction algorithms with methods that account for the role of structural waters would be vital to improving the accuracy of predicting novel water-supported structures.

In summary, we have determined several high-resolution crystal structures of the human monoclonal antibody 19b bound to HIV-1 Env V3 peptide. Our structures have provided atomic-level information on antibody-peptide contacts, including visualization of interfacial water molecules, and have revealed the structural basis for the recognition of V3 peptides from diverse isolates by 19b.

## MATERIALS AND METHODS

### 19b heavy and light chain sequences

#### 19b light chain

##### Nucleotide sequence

gctagcaccatggagacagacacactcctgctatgggtactgctgctctgggttccaggttccactggtgacgacattgtgctgacccagtctccaccctccctgtctgcatctgtcggagacagagtcaccatcacttgccaggcgagtcaggacattagcgaccatttaagttggtttcagcagaaaccagggaaagcccctaaactccttgtgtacggtgtatccagcttggaagcaggggtcccatctcggttcagtgtaagtggatctgggacacattttactttcaccgtcaacggcctgcagcctgaagatcttgcaacttatttctgtcagcagtatgatgatctcccgtggacgttcggcccagggaccgtggtggaagtcaaacgaactgtggctgcaccatctgtcttcatcttcccgccatctgatgagcagttgaaatctggaactgcctctgttgtgtgcctgctgaataacttctatcccagagaggccaaagtacagtggaaggtggataacgccctccaatcgggtaactcccaggagagtgtcacagagcaggacagcaaggacagcacctacagcctcagcagcaccctgacgctgagcaaagcagactacgagaaacacaaagtctacgcctgcgaagtcacccatcagggcctgagctcgcccgtcacaaagagcttcaacaggggagagtgttaggcggccgc

##### Amino acid sequence

metdtlllwvlllwvpgstgddivltqsppslsasvgdrvtitcqasqdisdhlswfqqkpgkapkllvygvssleagvpsrfsvsgsgthftftvnglqpedlatyfcqqyddlpwtfgpgtvvevkrtvaapsvfifppsdeqlksgtasvvcllnnfypreakvqwkvdnalqsgnsqesvteqdskdstyslsstltlskadyekhkvyacevthqglsspvtksfnrgec*

### 19b heavy chain

#### Nucleotide sequence

gctagcaccatggagacagacacactcctgctatgggtactgctgctctgggttccaggttccactggtgacgaggtgcagctggtacagtctgggggagccttgatccagccgggggggtccctgagactctcctgtgcagcctctggattcacttttgtcgattatgccatgagttgggtccgccaggctccagggaaggggctgcagtgggtctcaactattattggcagtggtgctgacacatactacacagactccgtgaagggccgcttcaccatctccagagacaattccaataatactgtgcatctgcaaatgaacagcctgcgagccgacgacacggccctctattattgtgtcagaggcgtctttgacctctggggccagggaacgctggtcaccgtctcctcagcctccaccaagggcccatcggtcttccccctggcaccctcctccaagagcacctctgggggcacagcggccctgggctgcctggtcaaggactacttccccgaaccggtgacggtgtcgtggaactcaggcgccctgaccagcggcgtgcacaccttcccggctgtcctacagtcctcaggactctactccctcagcagcgtggtgaccgtgccctccagcagcttgggcacccagacctacatctgcaacgtgaatcacaagcccagcaacaccaaggtggacaagagagttgagcccaaatcttgtgacaaaactcacacatgcccaccgtgcccagcacctgaactcctggggggaccgtcagtcttcctcttccccccaaaacccaaggacaccctcatgatctcccggacccctgaggtcacatgcgtggtggtggacgtgagccacgaagaccctgaggtcaagttcaactggtacgtggacggcgtggaggtgcataatgccaagacaaagccgcgggaggagcagtacaacagcacgtaccgtgtggtcagcgtcctcaccgtcctgcaccaggactggctgaatggcaaggagtacaagtgcaaggtctccaacaaagccctcccagcccccatcgagaaaaccatctccaaagccaaagggcagccccgagaaccacaggtgtacaccctgcccccatcccgggaggagatgaccaagaaccaggtcagcctgacctgcctggtcaaaggcttctatcccagcgacatcgccgtggagtgggagagcaatgggcagccggagaacaactacaagaccacgcctcccgtgctggactccgacggctccttcttcctctatagcaagctcaccgtggacaagagcaggtggcagcaggggaacgtcttctcatgctccgtgatgcatgaggctctgcacaaccactacacgcagaagagcctctccctgtctccgggtaaatgagcggccgc

#### Amino acid sequence

metdtlllwvlllwvpgstgdevqlvqsggaliqpggslrlscaasgftfvdyamswvrqapgkglqwvstiigsgadtyytdsvkgrftisrdnsnntvhlqmnslraddtalyycvrgvfdlwgqgtlvtvssastkgpsvfplapsskstsggtaalgclvkdyfpepvtvswnsgaltsgvhtfpavlqssglyslssvvtvpssslgtqtyicnvnhkpsntkvdkrvepkscdkthtcppcpapellggpsvflfppkpkdtlmisrtpevtcvvvdvshedpevkfnwyvdgvevhnaktkpreeqynstyrvvsvltvlhqdwlngkeykckvsnkalpapiektiskakgqprepqvytlppsreemtknqvsltclvkgfypsdiavewesngqpennykttppvldsdgsfflyskltvdksrwqqgnvfscsvmhealhnhytqkslslspgk*

### 19b expression and purification

Plasmids encoding the heavy and light chains of IgG 19b were mixed in a 1:1 molar ratio for transient transfection in Expi293i cells at 2.5 × 10^6 cells/mL followed by the addition of ExpiFectimine 18 hours later. Supernatant was harvested after 5 days and filtered at 0.22 μm. Supernatant was poured over Protein A resin using gravity chromatography. 19b was eluted using IgG Elution buffer (ThermoScientific) having adjusted to pH 7 using Tris pH 8. Antibody was concentrated to 35–73 mg/mL and stored at –80°C before Fab digest. LysC (Roche) was mixed in 1:2,000 mass ratio and reaction occurred at 37°C for 4 h. 1× protease inhibitor solution (Pierce) was added to stop the reaction and mixture stored at 4°C. A Protein A Plus Agarose NAb spin column was used to purify 19b Fab. Eluted Fab was concentrated and further purified by size-exclusion chromatography (Superose 6 Increase, 10/300 GL column) in 1× PBS pH 7.4, peak fractions were pooled and 19b Fab was concentrated to ∼50 mg/mL. Aliquots were snap cooled in liquid N2 and stored at – 80°C until use.

### SPR (surface plasmon resonance)

SPR binding measurements were performed at 25°C using a Cytiva Biacore T-200 instrument. V3 peptides with a biotin moiety at the C-terminus were diluted to 10 nM in HBS-EP^+^ buffer (10 mM HEPES pH 7.4, 3 mM EDTA, 0.005% P-20) and flowed over a high-affinity streptavidin (SA) sensor chip for capture with final response units (RUs) in the range of 50–200. 19b Fab was injected using the single-cycle kinetics mode with five concentrations per cycle in the range of: 20 nM, 10 nM, 5 nM, 2.5 nM, 1.25 nM, 0.625 nM, and 0.3125 nM. A contact time of 60 s and a dissociation time of 60 s were used at a flow rate of 50 µL/min. For 5768-p27 peptides, a d2 h dissociation time was required. The surface was regenerated after the last injection with three 10 s pulses at 100 µL/min of a 50 mM NaOH + 1 M NaCl solution, and for 5768(x)-p27 peptides, the solution was supplemented with 50% (vol/vol) ethylene glycol. Flow cell 1 was used as a reference. Blank sensorgrams were obtained by injection of the same volume of HBS-EP^+^ buffer in place of Fab solutions. Sensorgrams were reference-subtracted and corrected with corresponding blank curves. Sensogram data were analyzed using the BiaEvaluation software (Cytiva).

### DSF (differential scanning fluorimetry)

Samples were diluted to 1.25 mg/mL and run in triplicate on the Prometheus Panta (Nanotemper). Ramp was run at 1°C/min, from 25°C to 95°C with excitation at 280 nm and emission detected at 330 nm and 350 nm. PR.Panta Control v1.6.3 and PR.Panta Analysis v1.6.3 were used to collect and analyze the data, respectively.

### X-ray crystallography

19b Fab and V3 peptides were mixed in a 1:1.3 molar ratio as follows: for JR-FL, 92BR020, and 5768(x)-p27, 1.0 mg of the dried peptide powder was weighed, then dissolved in 50 mg/mL of 19b Fab in 1× PBS solution. Complexes were incubated for 1.5–3 h at 4°C. Unbound 19b Fab was defrosted for 1.5 h at 4°C before crystallization trials. Oryx4 (Douglass Instruments) and Mosquito (SPT Labtech) liquid handlers were used for the sitting drop vapor diffusion method. An Integra 300 was used to dispense 70 µL of the screening solutions onto the plates (Art-Robbins Intelliplate 3 well). Three drops were used for each condition: 0.2 μL protein + 0.2 µL condition, 0.15 μL protein + 0.25 µL condition, and 0.25 μL protein + 0.15 µL condition. Crystal Screen Cryo and PEG/Ion screens from Hampton were used to screen for initial crystals. Crystal growth was monitored by taking drop images using a Jansi UVEX robot imager in bright-field and UV. Crystals were harvested and cryoprotectant consisted of the condition supplemented with 30% (vol/vol) glycerol.

Details of X-ray diffraction data collection and indexing are provided in Table 1. Aimless was employed to create the .mtz file used for PHASER/MR in PHENIX, followed by PHENIX.refine. To create the 19b Fab structure, 17b Fab (PDB 7KLC) (38) was used as the initial Fab model. For initial model building, a single PDB file was created separating the variable region of the 17b light and heavy chains using PDB tools in PHENIX. The same was done for the constant region of the light and heavy chains of 17b. The model sequence was replaced with that of 19b (validated by GeneImmune). The V3 peptide model was built into the difference density initially for the 19b Fab - JR-FL complex, then modified for the other peptide structures. Omit maps were generated for the V3 peptides in complex with 19b. COOT v0.9.8.91,xf Pymol v2.2.3, PHENIX 1.20.1-4487 were used for model building, refinement, and figure making (39–41).

### AlphaFold modeling

Structural models of the 19b Fab in complex with HIV-1 Env V3 peptides were generated using the AlphaFold3 web server (https://alphafoldserver.com/). Predictions were performed using multi-chain heteromeric inputs comprising the Fab regions of the 19b heavy and light chains and the HIV-1 Env V3 peptide, with one copy of each chain specified. The amino acid sequences used for modeling correspond to those used experimentally for the 19b antibody and V3 peptides and were explicitly defined in the input configuration. Multiple input configurations were evaluated, including extended V3 peptide sequences (EINCTRPNNNTRKSIHIGPGRWFYTTGEIIGDIRQAHCNISRA) and a truncated construct (NNNTRKSIHIGPGRWFYTT) encompassing the epitope core. Independent prediction runs were performed using fixed random seeds to ensure reproducibility of stochastic elements. The highest-ranking predictions from both extended and truncated V3 constructs yielded consistent structural features; specifically, in all top-ranked models, 19b light chain residue Ser52 was not positioned within hydrogen-bonding distance of Arg315 in V3. A representative model generated with the truncated V3 construct is shown in Fig. S3. All other parameters, including multiple sequence alignment generation, template search, model inference, and confidence scoring, were handled using the default settings of the AlphaFold3 server. Because the server is continuously updated, the sequence databases, structural template libraries, and model weights correspond to those available at the time of job submission (14–16 October 2024).

## Data Availability

The structure coordinates were deposited to the PDB under accession codes 9MZ9, 9MMJ, 9MZ7, 9MZ6, and 9MZ8.

## SUPPLEMENTAL MATERIAL

Supplemental material jvi.02139-25-s0001.pdf Figures S1 to S3; Tables S1 to S4.

ASM does not own the copyrights to Supplemental Material that may be linked to, or accessed through, an article. The authors have granted ASM a non-exclusive, world-wide license to publish the Supplemental Material files. Please contact the corresponding author directly for reuse.
