# Supplementary material for: Structural determination of the HIV-1 Variable Region 3 epitope of antibody 19b
Source: J Virol. 2026 Jul 22;100(8):e02139-25. doi: 10.1128/jvi.02139-25 (PMC13483476; doi:10.1128/jvi.02139-25)
Supplement: Supplementary file 1 [file jvi.02139-25-s0001.pdf]

Figure S1

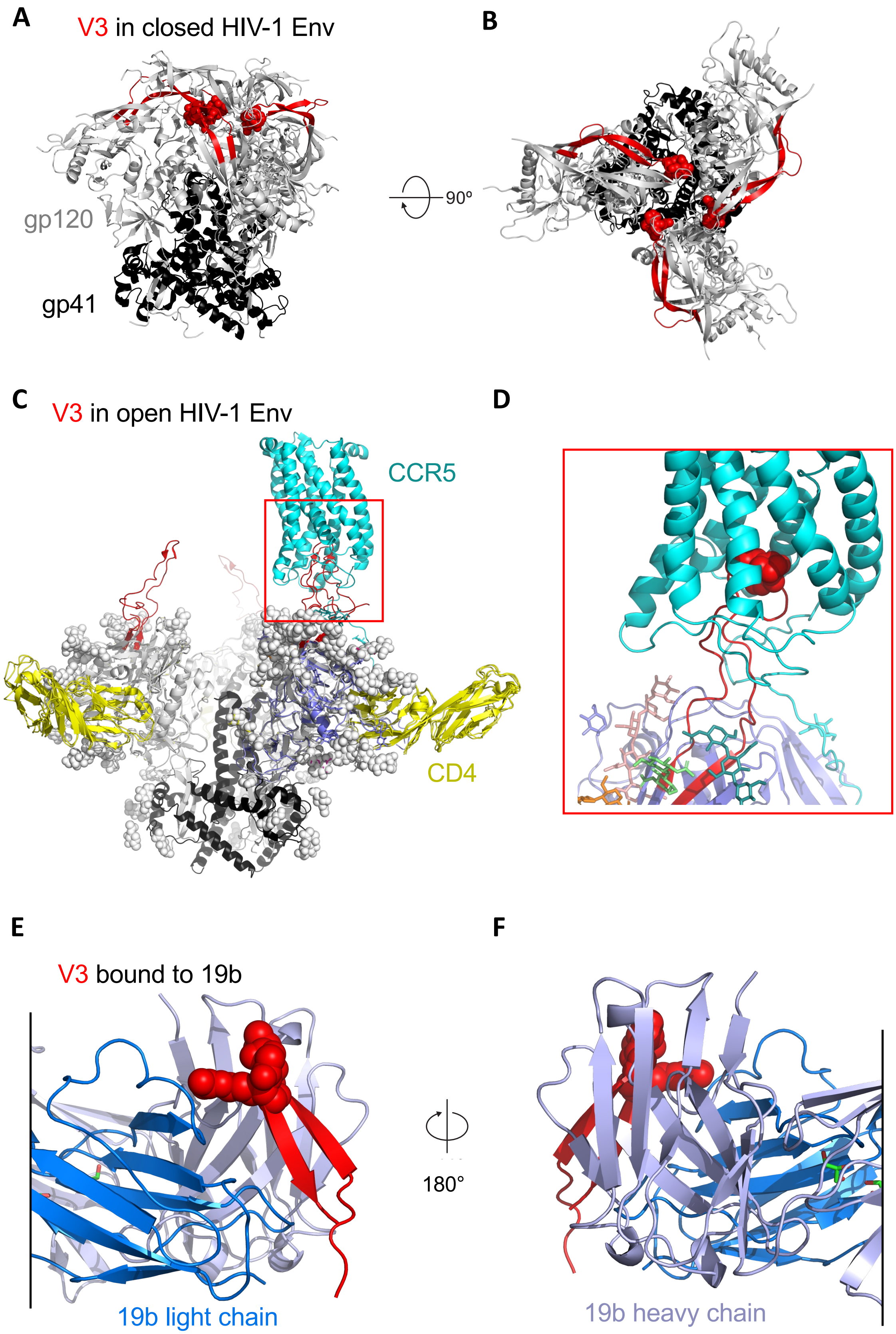
A
V3 in closed HIV-1 Env
gp120
gp41
B
90º
C
V3 in open HIV-1 Env
CCR5
CD4
D
E
V3 bound to 19b
19b light chain
180°
F
19b heavy chain

Figure S1

19b epitope in the context of the closed Env, open Env and CCR5-bound Env.

(A) Side view of closed Env (PDB: 5FYK) shown with gp120 colored light grey, gp41 black, and V3 red. The V3 GPGR motif is shown as spheres. This image is similar to the one in Figure 1A but shown here without the glycans to allow visualization of the buried V3 region. (B) 180° rotated view of panel A showing a view of the Env apex. (C) Side view of open Env shown with gp120 colored light grey, gp41 black, CD4 yellow, and V3 red. Glycans are shown as spheres. The model of the open Env was created from PDBs 5VN3, 5FYK, and 2B4C by superimposing the gp120 subunits for visualizing the open Env (PDB: 5VN3), with glycans (PDB: 5FYK) and the V3 (PDB: 2B4C). One protomer of the open Env is superimposed with the CD4/CCR5-bound gp120 (PDB: 6MEO), with CCR4 colored in cyan, its bound gp120 colored blue. (D) a zoomed-in view of the gp120/CCR5 interface. The V3 GPGR motif is shown as spheres. (E) V3 bound to 19b. (F) 90° rotated view of panel E. The V3 orientation in panel F is the same as in panel D.

Figure S2

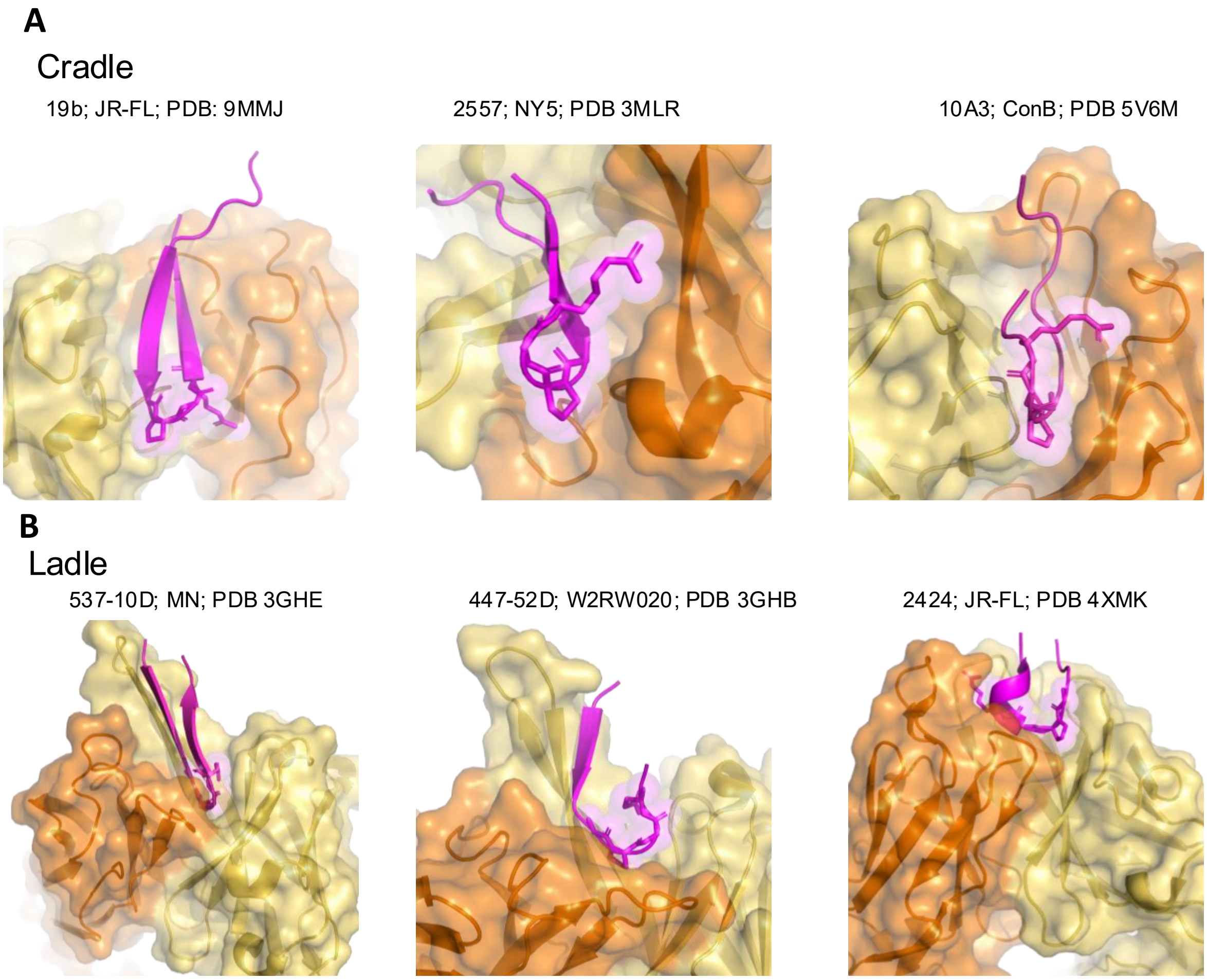

Figure S2
19b adopts the cradle binding mode
Representative examples of V3 antibodies that adopt the (A) cradle and (B) ladle binding mode.

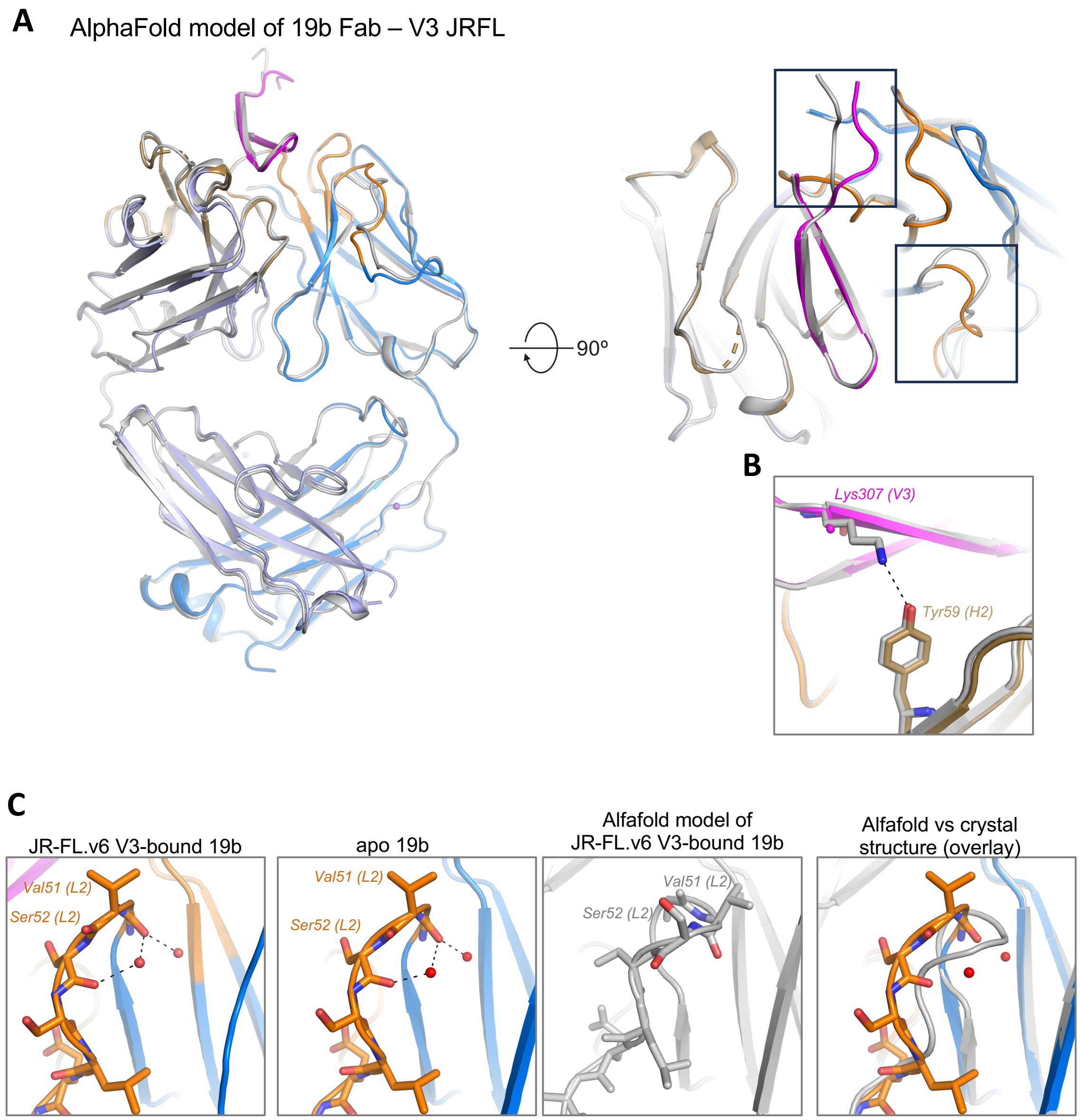

Figure S3

AlphaFold prediction of 19b crystal structures

(A) Superposition of 19b Fab – V3 JR-FL crystal structure with AlphaFold predicted model has Cα RMSD of 0.477 Å. AlphaFold model (grey) was predicted before deposit of the 19b crystal structures into the Protein Data Bank. (B) Zoomed in view of the V3 Lys307 – 19b Tyr59 (H2) hydrogen bond predicted. Side chain for Lys307 was not modeled in the crystal structure due to lack of side chain electron density. (C) Zoomed in views of the 19b CDR L2 loop for the Left to right. JR-FL.v6 V3-bound crystal structure, crystal structure of apo 19b Fab, AlphaFold predicted structure of JR-FL.v6 V3-bound 19b Fab complex, and overlay of the AlphaFold predicted and experimentally determined structures of the JR-FL.v6 V3-bound 19b Fab complex.

Table S1. Crystallographic data collection and refinement					
Crystal name	*19b Fab apo*	*19b Fab - V3 JR-FL*	*19b Fab - V3 92BR020*	*19b Fab - V3 5768-p27*	*19b Fab - V3 5768x-p27*
PDB ID	9MZ9	9MMJ	9MZ7	9MZ6	9MZ8
Data collection					
Space group	*P 21 21 21*	*P 21 21 21*	*P 21 21 21*	*P 21 21 21*	*P 21 21 21*
Unit-cell dimensions					
a, b, c (Å)	46.693 83.729 136.628	45.902 87.507 131.973	46.572 81.454 134.16	45.951 87.176 131.677	46.445 82.407 135.896
α, β, *Υ* (º)	90.0, 90.0, 90.0	90.0, 90.0, 90.0	90.0, 90.0, 90.0	90.0, 90.0, 90.0	90.0, 90.0, 90.0
Resolution (Å)	52.93 - 2.1 (2.15 - 2.1)	34.61 - 1.778 (1.82 - 1.78)	51.78 - 1.944 (1.99 - 1.94)	22.81 - 2.497 (2.56 - 2.5)	52.43 - 1.74 (1.78 - 1.74)
CC(1/2)	0.997 (0.551)	0.998 (0.889)	0.997 (0.767)	0.973 (0.522)	0.999 (0.483)
Rpim	0.03109 (0.4484)	0.028 (0.235)	0.048 (0.305)	0.137 (0.561)	0.034 (0.664)
Overall Rmerge	0.160 (1.929)	0.096 (0.719)	0.098 (0.605)	0.336 (1.232)	0.0114 (2.164)
I/σ(I)	11.09 (1.48)	26.0 (3.8)	11.0 (2.2)	11.34 (2.9)	16.9 (1.2)
Completeness (%)	99.94 (99.82)	99.8 (96.4)	98.6 (95.7)	99.72 (97.63)	99.9 (98.9)
No. reflection/unique	64,149 (32077)	57,058 (51,808)	75,265 (37,788)	37,915 (18,959)	108,759 (54,391)
Redundancy	12.7 (11.3)	6.6 (5.2)	5.8 (5.3)	8.2 (7.2)	13.1 (11.4)
Refinement					
R-work	0.2155 (0.3342)	0.1588 (0.1929)	0.2197 (0.2969)	0.1897 (0.2469)	0.1911 (0.2980)
R-free	0.2473 (0.3848)	0.1989 (0.2047)	0.2688 (0.3473)	0.2404 (0.2806)	0.2009 (0.2946)
Molprobity					
Ramachandran favored	96.43	96.98	95.81	94.90	95.55
Ramachandran allowed	3.57	2.55	3.72	4.64	3.98
Ramachandran outlier	0	0.46	0.47	0.46	0.47
Rotamer outliers	3.08	0.8	0.82	0	0.27
Clash score	2.72	3.26	4.51	4.21	2.71
No. of water	265	478	511	350	491
RMSD					
Bond lengths (Å)	0.002	0.012	0.003	0.003	0.009
Bond angles (°)	0.59	1.14	0.54	0.57	0.99

Table S2: 19b Fab crystallization conditions					
**PDB code**	9MZ9	9MMJ	9MZ7	9MZ6	9MZ8
**V3 peptide**	N/A	JRFLv6	92BR020	5768-p27	5768x-p27
**Peptide synthesized**	N/A	EIncTRPNNNTRKSIHIGPGRWFYTTGEIIGDIRQAHcnISRA	EIncTRPNNNTRKSIHIGPGRAFYATGDIIGDIRQAHcnISRA	EIncTRPNNNTRKSIHMGPGKVFYTTGEIIGDIRQAHcnISRA	EIncTRPNNNTRKSIHxGPGKVFYTTGEIIGDIRQAHcnISRA
**V3 residues with electron density to build model**	N/A	NNNTRKSIHIGPGRWFYTT	RKSIHIGPGRAFYAT	NNNTRKSIHMGPGKVFYTT	RKSIHXGPGKVFYTTG
**Peptide residues in structure**	N/A	19	15	19	16
**X-ray source**	LBL ALS 5.0.1	BNL 19-ID NYX	LBL ALS 5.0.1	Bruker Venture D8, University of North Carolina at Chapel Hill	LBL ALS 5.0.1
**X-ray indexing**	XDS	HKL2000	XDS	Saint for integration, SADABS for scaling	XDS
**Crystallization condition**	0.2 M Magnesium acetate tetrahydrate, 20% w/v Polyethylene glycol 3,350, pH 7.9 (PEG/Ion C1)	0.18 M Ammonium sulfate, 0.09 M Sodium acetate trihydrate pH 4.6, 27% w/v Polyethylene glycol monomethyl ether 2,000, 10% v/v Glycerol (Crystal Screen Cryo, F1)	0.18 M Ammonium sulfate, 0.09 M Sodium acetate trihydrate pH 4.6, 27% w/v Polyethylene glycol monomethyl ether 2,000 , 10% v/v glycerol (Crystal Screen Cryo, F1)	0.17 M Ammonium sulfate, 0.085 M Sodium cacodylate trihydrate pH 6.5, 25.5% w/v Polyethylene glycol 8,000, 15% v/v Glycerol (Crystal Screen Cryo, B3)	0.2 M Lithium sulfate monohydrate, 20% w/v Polyethylene glycol 3,350, pH 6.0 (PEG/Ion, C7)
**19b Fab concentration (A280)**	49 mg/mL	53 mg/mL	49 mg/mL	49 mg/mL	54 mg/mL
**19b Fab:V3 peptide molar ratio**	N/A	1:1.3	1:1.3	1:1.3	1:1.3

Table S3: RMSDs of 19b Fab structures compared to 19b Fab - V3 JRFL										
**Peptide**	**PDB Code**	**Light Chain**	**Heavy Chain**	**CDR L1**	**CDR L2**	**CDR L3**	**CDR H1**	**CDR H2**	**CDR H3**	**V3**
Ligand-free	9MZ9	0.453	1.039	0.094	0.143	0.117	0.069	0.121	0.295	N/A
92BR020	9MZ7	0.478	0.517	0.087	0.143	0.075	0.119	0.104	0.104	0.208
5768-p27	9MZ6	0.144	0.147	0.097	0.113	0.079	0.106	0.083	0.111	0.201
5768x-p27	9MZ8	0.44	0.983	0.087	0.189	0.041	0.091	0.062	0.066	0.202

Table S4: Interactions of 19b with V3 peptides from different HIV-1 isolates

Heavy chain interactions

NAME	##	Structure 1	HSDC	ASA	BSA
19b Fab with V3 JR-FL peptide	8	D:ILE 309		80.05	58.1
	9	D:HIS 310		111.94	23.53
	10	D:ILE 311	H	96.4	89.97
	11	D:GLY 312		18.13	15.13
	12	D:PRO 313		135.35	59.68
	14	D:ARG 315		183.23	42.67
	15	D:TRP 316		117.77	2.14
	16	D:PHE 317		135.33	56.55
	18	D:THR 319		64.64	6.02
	1	D:ASN 302		208.06	20.46
	2	D:ASN 303	H	108.08	40.02
	4	D:THR 305		99.55	9.57
19b Fab with V3 92BR020 peptide	4	D:ILE 309		83.62	61.54
	5	D:HIS 310		139.53	26.39
	6	D:ILE 311	H	93.3	86.03
	7	D:GLY 312		18.01	15.52
	8	D:PRO 313		135.33	58.02
	10	D:ARG 315		188.12	41.49
	11	D:ALA 316		32.92	1.02
	12	D:PHE 317		133.21	53.73
	14	D:ALA 319		46.83	3.52
19b Fab with V3 5768-p27 peptide	8	D:ILE 309		84.78	62.84
	9	D:HIS 310		133.26	31.37
	10	D:MET 311	H	108.74	100.18
	11	D:GLY 312		17.32	14.14
	12	D:PRO 313		131.98	57.8
	14	D:LYS 315		150.69	35.4
	15	D:VAL 316		54.45	2.12
	16	D:PHE 317		128.82	49.28
	18	D:THR 319		61.21	5.02
	1	D:ASN 302		203.26	22.21
	2	D:ASN 303		106.16	46.4
	4	D:THR 305		98.1	7.98
19b Fab with V3 5768x-p27 peptide	4	D:ILE 309		84.62	61.52
	5	D:HIS 310		134.27	31.55
	6	D:NLE 311	H	102.99	95.49
	7	D:GLY 312		19.47	16.46
	8	D:PRO 313		131.61	57.13
	10	D:LYS 315		145.89	40.52
	11	D:VAL 316		56.63	1.51
	12	D:PHE 317		131.58	50.47
	14	D:THR 319		60.86	6.19

Light chain interactions

NAME	##	Structure 1	HSDC	ASA	BSA
19b Fab with V3 JR-FL peptide	5	D:ARG 306	HS	122.37	60.47
	8	D:ILE 309		80.05	21.96
	10	D:ILE 311		96.4	6.43
	14	D:ARG 315	HS	183.23	113.73
	15	D:TRP 316	H	117.77	18
	16	D:PHE 317		135.33	78.77
	17	D:TYR 318	H	130.85	89.18
	18	D:THR 319	H	64.64	43.88
	19	D:THR 320	H	129.18	22.16
19b Fab with V3 92BR020 peptide	1	D:ARG 306	HS	216.03	70.48
	4	D:ILE 309		83.62	20.82
	6	D:ILE 311		93.3	7.28
	10	D:ARG 315	HS	188.12	117.41
	11	D:ALA 316	H	32.92	16.46
	12	D:PHE 317		133.21	79.48
	13	D:TYR 318	H	136.16	91.05
	14	D:ALA 319	H	46.83	36.55
	15	D:THR 320	H	145.78	19.74
19b Fab with V3 5768-p27 peptide	5	D:ARG 306	HS	119.05	58.52
	8	D:ILE 309		84.78	21.94
	10	D:MET 311		108.74	8.56
	13	D:GLY 314		81.13	0.24
	14	D:LYS 315		150.69	77.73
	15	D:VAL 316	H	54.45	18.42
	16	D:PHE 317		128.82	79.55
	17	D:TYR 318	H	144.65	91.42
	18	D:THR 319	H	61.21	41.27
	19	D:THR 320	H	130.52	23.22
	1	D:ASN 302	H	203.26	79.83
	2	D:ASN 303		106.16	5.91
	3	D:ASN 304	H	145.85	53.42
19b Fab with V3 5768x-p27 peptide	1	D:ARG 306	HS	193.92	53.52
	4	D:ILE 309		84.62	23.11
	6	D:NLE 311		102.99	7.5
	9	D:GLY 314		80.34	0.49
	10	D:LYS 315		145.89	74.54
	11	D:VAL 316	H	56.63	20.08
	12	D:PHE 317		131.58	81.11
	13	D:TYR 318	H	134.83	92.24
	14	D:THR 319	H	60.86	41.25
	15	D:THR 320	H	85.7	18.35

Analysis was performed using PDBePisa (https://www.ebi.ac.uk/pdbe/pisa/).
HSDC: Hydrogen bonds/Salt bridges/Difulfide links/Covalent links
ASA: Accessible surface area
BSA: Buried Surface Area
